# Comment on “Ambient Coarse Particulate Matter and Hospital Admissions in the Medicare Cohort Air Pollution Study, 1999–2010”

**DOI:** 10.1289/ehp.1510123

**Published:** 2015-09-01

**Authors:** Eduardo Hernández-Garduño

**Affiliations:** Instituto de Seguridad Social del Estado de México y Municipios (ISSEMyM)

In a national-level study of elderly people, Powell et al. found that a 10-µg/m^3^ increase in coarse particulate matter (PM_10–2.5_) was associated with a statistically significant increase of 0.69% (95% posterior interval [PI]: 0.45, 0.92) in cardiovascular hospitalizations on the same day as exposure. The cause-specific analysis showed that the greatest association was found for heart rhythm disturbances, with an increase of 0.94% (95% PI: 0.40, 1.48), followed by ischemic heart disease at 0.74% (95% PI: 0.29, 1.20) and cerebrovascular disease at 0.72% (95% PI: 0.22, 1.21). A 10-µg/m^3^ increase in PM_2.5_ has also been recently associated with a 0.68% increase in out-of-hospital coronary deaths on the same day (Dai et al. 2015) and with ischemic heart disease mortality (hazard ratio = 1.19; 95% confidence interval [CI]: 1.08, 1.31) in a study assessing the chronic effect of particles in more than 100,000 women (Ostro et al. 2015).

In the study by Powell et al., respiratory hospitalizations overall and chronic obstructive pulmonary disease (COPD) in particular were not significantly associated with PM_10–2.5_ exposure. However, after adjusting for PM_10–2.5_, a 10-µg/m^3^ increase in PM_2.5_ was associated with a significant increase in respiratory hospitalizations on the same day (0.67%; 95% PI: 0.14, 1.21). A study by Qiu et al. (2012) showed that after adjusting for PM_2.5_ in two-pollutant models, estimated effects of PM_10–2.5_ on respiratory and COPD hospital admissions were attenuated but remained statistically significant, with excess relative risks of 1.05% (95% CI: 0.19, 1.91) and 1.78% (95% CI: 0.41, 3.16), respectively. All the aforementioned studies focused not only on the broad categories of cardiorespiratory diseases but also on cause-specific cardiorespiratory hospitalizations or mortality, and they confirmed once more that particulate matter is the air pollutant most consistently associated with these end points.

Recent climate change studies also have shown an association between weather variables and cardiorespiratory mortality. A recent multicounty/multicity study in Northeast Asia by Chung et al. (2015) found that extreme ambient temperatures were associated with cardiorespiratory mortality after adjusting for atmospheric pressure, relative humidity, and air pollution data. An interesting finding in this study was the decrease of cold effect on mortality by 2.36% (95% CI: −4.27, −0.45) associated with an increase in the interquartile range of annual average daily mean atmospheric pressure.

The effect of barometric pressure was not assessed by Powell et al. or any of the other aforementioned authors, with the exception of Chung et al. (2015). There is evidence of a positive correlation between barometric pressure and blood oxygen saturation in the elderly, suggesting that barometric pressure may produce physiologic changes (Pope et al. 1999). For this reason, barometric pressure should always be considered in studies of air pollution and cardiorespiratory hospitalization/mortality to evaluate whether there is confounding or effect modification of the air pollutant estimates. Further studies measuring the effect of barometric pressure are warranted, particularly in populations of patients susceptible to changes in blood oxygen saturation or those undergoing long-term oxygen therapy, including patients with heart rhythm disturbances, ischemic heart disease, heart failure, associated respiratory failure in patients with interstitial lung disease, COPD, etc. Because barometric pressure changes with altitude, multicity studies would determine whether its effect differs by location.
